# Global trends and current status of distraction osteogenesis: Bibliometric analysis of publications from 1980 to 2021

**DOI:** 10.3389/fbioe.2022.1046476

**Published:** 2022-11-02

**Authors:** Qi Liu, Jieyu Liang, Ze Liu, Hongbin Guo, Min Wang, Yi Zhang

**Affiliations:** ^1^ Department of Orthopaedics, Xiangya Hospital, Central South University, Changsha, Hunan, China; ^2^ National Clinical Research Center for Geriatric Disorders, Xiangya Hospital, Central South University, Changsha, Hunan, China; ^3^ Department of Endocrinology, Xiangya Hospital, Central South University, Changsha, Hunan, China

**Keywords:** distraction osteogenesis, bibliometrics, web of science, VOSviewer, visual analyses

## Abstract

**Introduction:** Distraction osteogenesis (DO) has become an important technology for the correction of various congenital and acquired skeletal ridge deformities. It is widely used in oral and maxillofacial surgery, orthopedics, and other disciplines. From 1980 to 2021, the cutting-edge research of DO has been continuously promoted, and the interaction between disciplines has also been deepening. However, the analysis on the global trend and status of DO is relatively rare. Therefore, the aim of our study was to summarize the global trends and current status of DO through bibliometrics.

**Materials and methods:** Web of Science (WOS) core collection database and Medline were used to search DO-related literatures published during 1980–2021. The collected data are imported into Microsoft Excel, Microsoft Word, VOSviewer software for analysis and drawing figure/table.

**Results:** A total of 7,721 publications were included in this study. The United States is the main contributing country to DO (ranking first in terms of total publications, sum of times cited and H-index). Harvard University was the main contributing institution to DO. Journal of Craniofacial Surgery is the main contributing journal of DO related articles. Buchman, SR is the main contributing author to DO related articles. DO related publications can be summarized into 7 clusters: 1) “mechanism study”, 2) “limb bone distraction study”, 3) “alveolar bone distraction study”, 4) “temporomandibular joint ankylosis study”, 5) “maxillofacial surgery study”, 6) “skull distraction study” and 7) “mandible distraction study”. Mandible distraction study has been a hot topic in recent years. In addition, the “management”, “osteogenesis” and “reconstruction” of DO have been the research hotspots from 1980 to 2021.

**Conclusion:** From 1980 to 2021, the total number of DO articles has increased rapidly and maintained a steady trend. The United States is the predominant country in the field. Surgery, dental, and oral surgery and orthopaedics are hot fields of DO research. The study of mandible distraction has been paid more and more attention and will become a hotspot in the future. Our study is beneficial for scientists to specify the research hotspot and development direction of DO.

## 1 Introduction

Distraction osteogenesis (DO) was first used by Codivilla (1905) to lengthen the femur axially (Codivilla, 2008). The principle of DO is that the regenerative signal system of the body is activated under the action of continuous, stable and slow pulling force, which stimulates the division and regeneration of tissue cells, and the bone tissue. The attached muscles, fascia, blood vessels, nerves, and skin will grow synchronously. In the 1950s, Professor Ilizarov, a physician of the former Soviet Union, used DO to correct long bone defects and deformities of limbs, and developed the technology into mature (Liu et al., 2022). In 1992, McCarthy et al. (1992a) first used DO technique to correct hemifacial micrognathia and Nagers’ syndrome. Since then, DO technique has been widely used to correct various congenital and acquired maxillofacial deformities.

From 1980 to 2021, more and more publications have reported the clinical application of DO in orthopaedics, oral and maxillofacial surgery and neurosurgery around the world (Sandhaus and Johnson, 2021; Shevtsov and Leonchuk, 2021; Kosyk et al., 2022). However, the bibliometric analysis on the global trend and status of DO is relatively rare. Bibliometrics is a cross-discipline that uses mathematical and statistical methods to quantitatively analyze knowledge carriers, which can effectively avoid the subjectivity and arbitrariness of literature analysis and improve the formality and credibility of the results (Mayr and Scharnhorst, 2015). Through the systematic arrangement and reflection of literature, it is helpful for scholars to find a research breakthrough, and guide the future research direction. Therefore, the purpose of this study is to analyze the DO-related publications from 1980 to 2021 by bibliometrics, and to study the country, institutions, journal and authors in the field of DO. The results of our study may be helpful to facilitate the communication and cooperation among researchers, and then grasp the research trends in this field.

## 2 Materials and methods

### 2.1 Data sources

**FIGURE 1:**
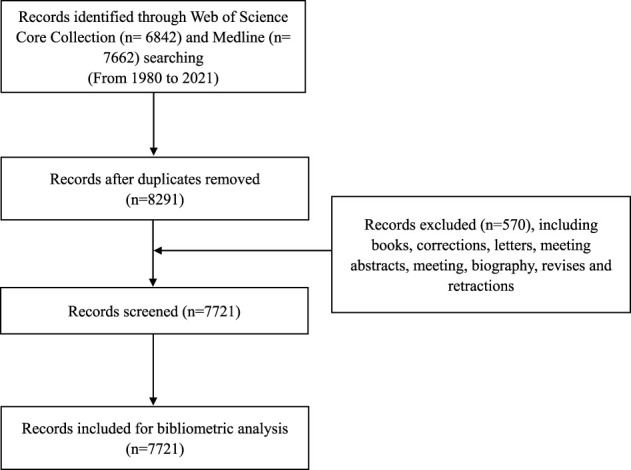
Flow chart of data collection in our bibliometric analysis.

All publications were sourced from Web of Science (WOS) Core Collection database (SCI-Expanded, SSCI, A&HCI, CPCI-S, CPCI-SSH, ESCI, CCR-Expanded, and IC) and Medline (Figure 1).

### 2.2 Search strategy

All publications were searched in WOS on October 2022. The search terms were TS = (“distraction osteogenesis” OR “bone lengthening”) and document types: (Article OR Review). We selected publications with publication dates ranging from 1 January 1980 to 31 December 2021.

### 2.3 Data collection

The full records (including title, publication year, author nationalities, institutions of authors, funding sources, journals of publications, abstracts, keywords, total number of publications, sum times of cited, average citations per item, and H-index) were extracted from the retrieved literature by two independent authors (QL and YZ). The disagreements were resolved by discussions to prevent potential bias. The obtained publication information was exported in TXT format, and then imported into Microsoft Excel 2019 and VOSviewer (v.1.6.18) for analysis.

### 2.4 Bibliometric analysis

Microsoft Excel 2019 was used to analyze literature data and draw graphs. Bibliometric indicators, including total publications, sum of times cited, average citations per item, H-index and self-citation times, were included in this study. Total publications are widely used to measure the contribution to a field. Sum of times cited and average citations per item reflects the level of attention (Taubes, 1993). The H-index reflects the number and quality of an author’s publications, which means that a scholar has published H papers, each of which has been cited at least H times by other publications (Roldan-Valadez et al., 2019; Lu et al., 2022). In addition, it can now also define the publication quality of a country/region, institution or journal (Noruzi et al., 2022).

VOSviewer is one of the much scientific knowledge graph software, which can demonstrate the influence, cooperation and evolution of research field through bibliographic coupling analysis, co-authorship analysis, co-citation analysis and co-occurrence analysis. Bibliographic coupling analysis is a way of showing similar relationships between items by the number of references co-cited by items. Co-authorship analysis is a way to assess the intensity of collaboration between items by counting the number of co-authored publications. Co-citation analysis is a way of presenting the relevance of items based on how many times an item is cited together. Co-occurrence network visualization is created by analyzing the number of publications in which keywords appear together in the title or abstract. The aim is to identify hot research directions and topics that are essential to tracking scientific developments (Wang et al., 2019). In some way, the total link strength (TLS) can measure the degree of influence, collaboration of the items as a bibliometric index. In network visualization, the circle and label form items. The larger the circle, the more the number of publications. The thickness of line represents the correlation strength, and the circle color represents different cluster.

## 3 Results and discussion

### 3.1 Trend analysis of distraction osteogenesis-related publications

**FIGURE 2:**
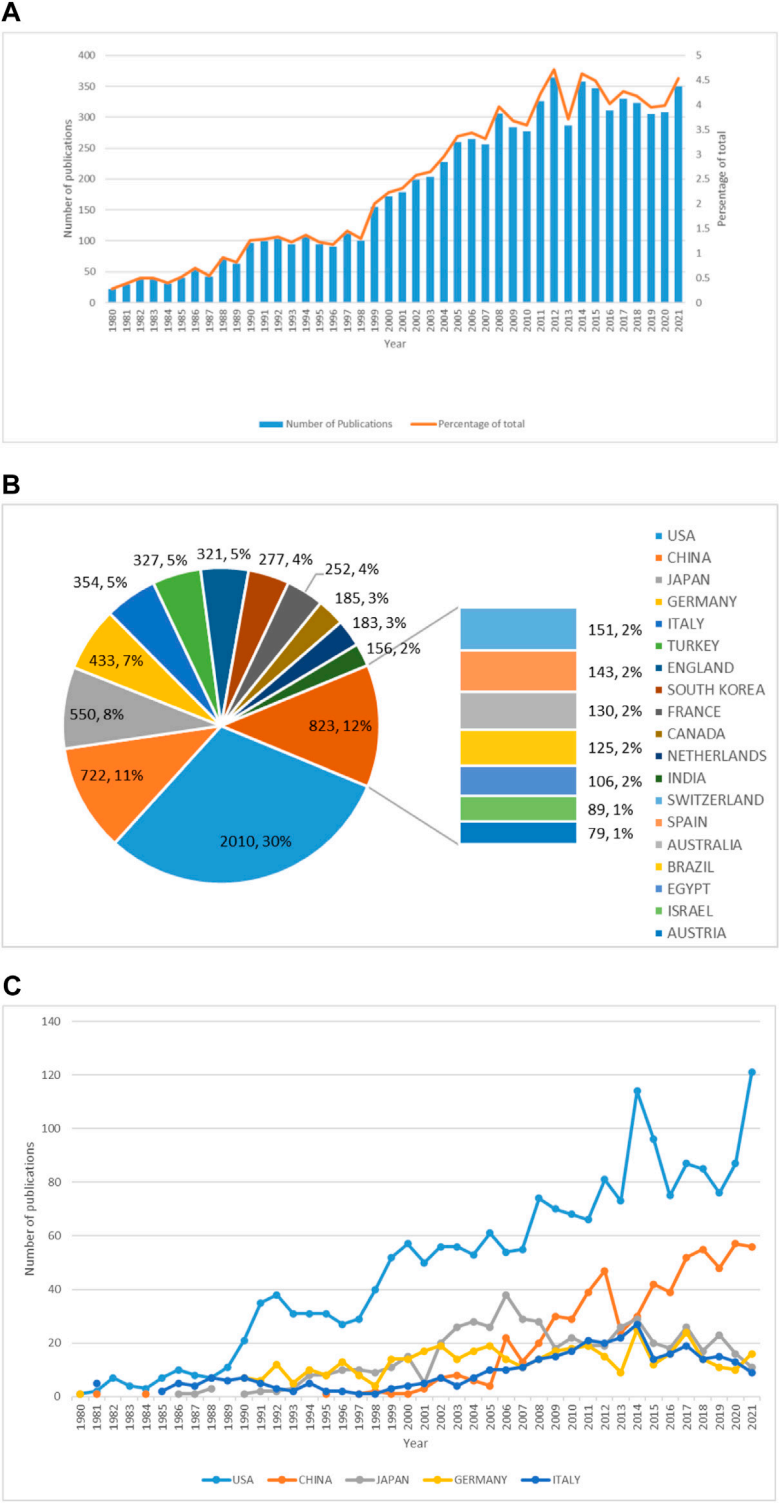
Global trends of publications on distraction osteogenesis (DO) in the last 42 years. **(A)** Annual DO-related publications worldwide. **(B)** The total number and percentage of DO-related publications from the top twenty countries. **(C)** DO-related publications of the top five countries over time.

Our study identified 7721 DO-related publications, including article 6685) and review (1036), published between 1980 and 2021. The annual publications related to DO have maintained a steady trend after rapid growth. During 1980 to 2021, the number of annual publications on DO increased fifteenfold, from 22 in 1980 to 350 in 2021 (Figure 2A). It showed a steady upward trend from 1980 to 2012, and the publication volume tended to be stable from 2013 to 2021. A total of 99 countries participated in the DO study, and Figure 2B shows the top 20 countries. Among them, The United States ranks first in total publications (2010, 30%), followed by China (722, 11%), Japan (550, 8%), Germany (433, 7%), and Italy (354, 5%). In addition, among the top 5 countries, the United States and China showed an upward trend year by year, while the number of publications issued in other countries did not increase significantly (Figure 2C).

As shown in this study, from 1980 to 2021, the number of DO-related publications peaked in 2012 and then leveled off. We can predict that there will be more than 250 DO-related publications per year in the next few years. In addition, DO-related publications in the United States and China will continue to increase. From the fan chart, we can see that DO related researchers come from all over the world, especially in the United States, China, and Japan.

### 3.2 Quality analysis of distraction osteogenesis-related publications

#### 3.2.1 Country

**FIGURE 3:**
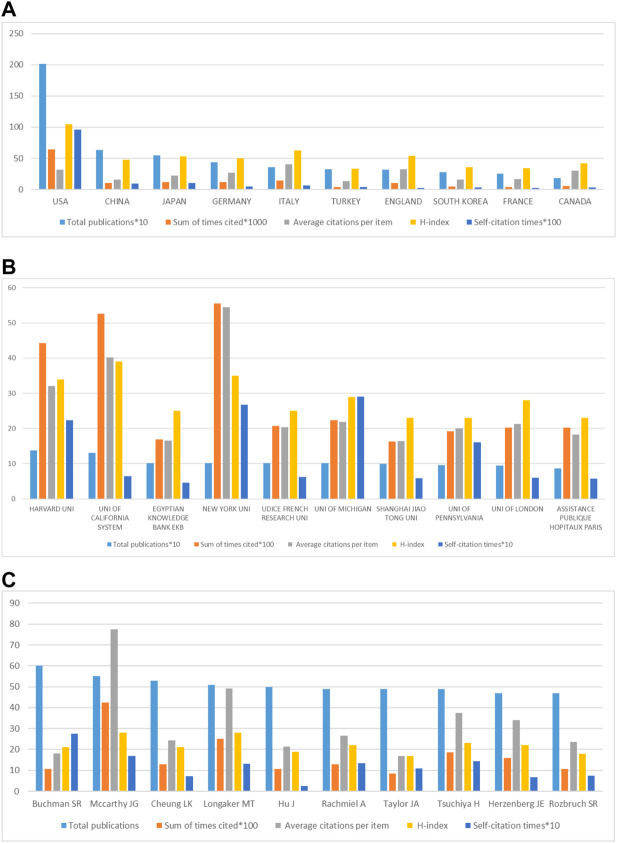
Quality analysis of global publications in DO in the last 42 years. **(A)** Total publications, sum of times cited, average citations per item, H-index and self-citation times of the top ten countries by contributions. **(B)** Total publications, sum of times cited, average citations per item, H-index and self-citation times of the top ten institutions by contributions. **(C)** Total publications, sum of times cited, average citations per item, H-index and self-citation times of the top ten authors by contributions.

Figure 3A shows the quality measurement indicators (including sum times for cited, the average times of citations per item, H-index and self-citation times) of the top 10 DO-related publications. Among the top 10 countries in the total number of publications, the United States ranked first, has higher sum times of cited (64436) and H-index (105) than the other 9 countries. Although China ranked second in total publications, it ranked 9th in the average times of citations per item (15.87), slightly ahead of Turkey (13.42). Other developed countries, including Italy (40.23), England (32.88), Canada (30.57), Germany (27.16), Japan (21.87), France (17.09), and South Korea (16.25), despite the small total publications, the average times of citations per item is relatively high, of which Italy ranks first in the world. The above studies suggested that the quality of publications in the United States is comparatively high, while those in China are relatively low. The reason for the situation maybe that China’s past scientific evaluation system paid more attention to the quantity rather than the quality of publications.

#### 3.2.2 Institution

From 1980 to 2021, about 5742 institutions around the world have studied DO. Figure 3B shows the top 10 global main contributing institutions. Among the 10 institutions, there are 5 in the United States (Harvard University, University of California, New York University, University of Michigan, University of Pennsylvania), 2 in France (Udice French Research Universities, Assistance Publique Hopitaux Paris), 1 in China (Shanghai Jiaotong University), 1 in Egypt (Egyptian Knowledge Bank) and 1 in England (University of London), respectively. Harvard University ranks No. 1 in the total publication of DO research, which has published 138 papers. The University of California ranked second (131 publications), followed by Egyptian Knowledge Bank (102 publications), New York University (102 publications), and Udice French Research Universities (102 publications). The above data show that Harvard University is the most contributing institution in the world.

#### 3.2.3 Author

The top 10 main contributing authors to the analysis of the quality of the author’s publication are shown in Figure 3C. Among these authors, seven are from the United States (Buchman SR, Mccarthy JG, Longaker MT, Rachmiel A, Taylor JA, Herzenberg JE, and Rozbruch SR), two from China (Cheung LK and Hu J), one from Japan (Tsuchiya H). In addition, two from the University of Michigan (Buchman SR, Rachmiel A), one from the Children’s Hospital of Philadelphia (Taylor JA), Mccarthy JG from New York University, Cheung LK from the University of Hong Kong, Longaker MT from Stanford University, Hu J from Sichuan University, Tsuchiya, H from Kanazawa University, Herzenberg JE from Sinai Hospital of Baltimore, Rozbruch SR from Cornell University. The biggest contributing authors is Buchman SR, which has published 60 papers with 1081 citations, with average citations per item of 18.02 and a H-index of 21. Mccarthy JG (55 articles, 4256 citations, average citations of 18.02, H-Index 28), which ranks second in the total number of publications, ranks first in the number of citations, and his H-index ranks first with Longaker MT. The above data show that Buchman SR is the author of DO-related publications with the greatest contribution. Interestingly, most of the authors with high contributions come from countries and institutions with high contributions, which shows that top research platforms can enable authors to create more contributions.

#### 3.2.4 Journal

**FIGURE 4:**
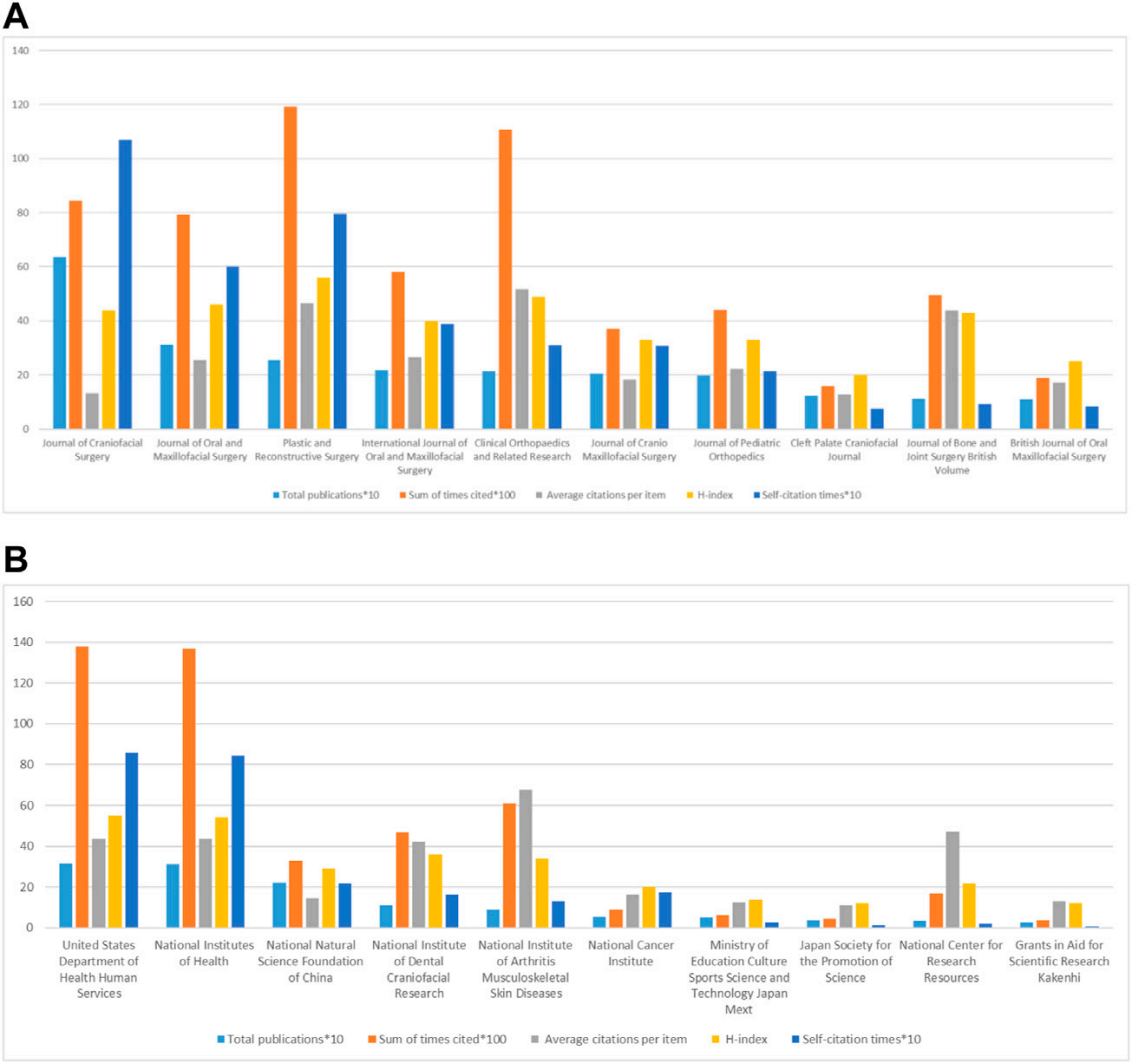
Analysis of highly contributing journals and funding agencies in DO in the last 42 years. **(A)** Total publications, sum of times cited, average citations per item, H-index and self-citation times of the top ten journals. **(B)** Total publications, sum of times cited, average citations per item, H-index and self-citation times of the top ten funding agencies.

Figure 4A shows the top 10 journals that have published the most DO-related publications. Journal of Craniofacial Surgery (IF = 1.172, 2022) is the largest number of DO-related publications (635 publications), followed by Journal of Oral and Maxillofacial Surgery (IF = 2.136, 2022) with 311 publications, Plastic and Reconstructive Surgery (IF = 5.169, 2022) with 256 publications, International Journal of Oral and Maxillofacial Surgery (IF = 2.986, 2022) with 219 publications, Clinical Orthopaedics and Related Research (IF = 4.755, 2022) with 214 publications.

Among the top 10 journals, Journal of Craniofacial Surgery rank first, which is the journal with the largest contribution to DO-related publications. In addition, Plastic and Reconstructive Surgery ranked third, ranking first in the sum of citations and H-index. The higher average citations per item indicate that a higher level of attention for its publication (Garcia et al., 2021). However, journals’ excessive self-citation can artificially affect journal metrics (Sanfilippo et al., 2021). We found that Journal of Craniofacial Surgery had a relatively high self-citation rate (12.6%), while the remaining nine journals were all below 10%. The above shows that Plastic and Reconstructive Surgery and Clinical Orthopaedics and Related Research are journals with high publication quality.

#### 3.2.5 Funding agency

Figure 4B presents the top 10 funding organizations of the largest of DO-related publications. The top funding agency in the number of publications is the United States Department of Health and Human Services (HHS, United States), followed by the National Institutes of Health (NIH, United States), the National Natural Science Foundation of China (NSFC, China), which indicates that these institutions made a significant contribution to DO-related publications. The higher average citations per item funding agencies are National Institute of Arthritis Musculoskeletal Skin Diseases (NIAMS, United States), National Center for Research Resources and the United States Department of Health and Human Services (HHS, United States), which indicates that the publications funded by these funding agencies are of high quality.

### 3.3 Analysis of highly cited distraction osteogenesis-related publications

Table 1 indicates the top 10 most cited DO-related publications with citations ranging from 520 to 1566. McCarthy et al. (1992b), Buser et al. (2004), Aghaloo and Moy (2007) wrote the first, sixth, eighth most cited publications, respectively. They all made a comprehensive summary and analysis of the anatomy, treatment plan and matters needing attention of DO operation of oral and maxillofacial bone. Ilizarov (1989a) and Ilizarov (1990), Paley (1990) wrote the second and 10th, fifth most cited publications, respectively. They detailed the best strategy and complications of DO operation of limb bone. Di Martino et al. (2005), Dimitriou et al. (2011), Claes et al. (2012), Carter et al. (1998) wrote the third, fourth, seventh, and ninth most cited publications, respectively. They summarized and studied the therapeutic strategies, influencing factors and mechanobiology mechanisms of promoting bone regeneration in the process of DO.

**TABLE 1:** Top ten most cited publications in DO in the world.

Title	Authors	Journal	Year	Type	IF	Times cited
Lengthening the human mandible by gradual distraction	McCarthy JG et al	Plastic and reconstructive surgery	1992	Article	5.169	1566
The tension-stress effect on the genesis and growth of tissues: Part II. The influence of the rate and frequency of distraction	Ilizarov GA	Clinical orthopaedics and related research	1989	Article	4.755	1411
Chitosan: A versatile biopolymer for orthopaedic tissue-engineering	Di Martino A et al	Biomaterials	2005	Review	2.69	1255
Bone regeneration: current concepts and future directions	Dimitriou, R et al	BMC medicine	2011	Review	11.15	1032
Problems, obstacles, and complications of limb lengthening by the Ilizarov technique	Paley D	Clinical orthopaedics and related research	1990	Article	4.755	1024
Optimizing esthetics for implant restorations in the anterior maxilla: Anatomic and surgical considerations	Buser D et al	International journal of oral and maxillofacial implants	2004	Article	2.912	706
Fracture healing under healthy and inflammatory conditions	Claes L et al	Nature reviews rheumatology	2012	Review	32.286	660
Which hard tissue augmentation techniques are the most successful in furnishing bony support for implant placement?	Aghaloo T et al	International journal of oral and maxillofacial implants	2007	Review	2.912	620
Mechanobiology of skeletal regeneration	Carter DR et al	Clinical orthopaedics and related research	1998	Article	4.755	526
Clinical application of the tension-stress effect for limb lengthening	Ilizarov GA	Clinical orthopaedics and related research	1990	Article	4.755	520

### 3.4 Analysis of distraction osteogenesis-related research areas

**FIGURE 5:**
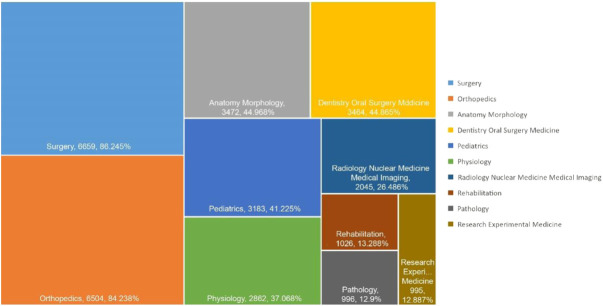
Research area analysis of global publications in DO in the last 42 years.

DO-related publications are divided into 118 different research fields on WOS. We analyzed the top 10 research areas of DO-related publications. Figure 5 shows the corresponding number of DO-related publications in all research areas, among which surgery area receiving the most attention (6659, 86.245%), followed by orthopaedics area (6504,84.238%), anatomy morphology area (3472,44.968%), dentistry oral surgery medicine area (3464,44.865%), pediatrics area (3183,41.225%), physiology area (2862,37.068%), radiology nuclear medicine medical imaging area (2045,26.486%), rehabilitation area (1026,13.288%), pathology area (996,12.9%), and research experimental medicine area (995,12.887%).

Surgery, dentistry oral surgery medicine, orthopaedics and radiology nuclear medicine medical imaging have been widely and deeply studied for their attention to the influencing factors, surgical methods, diagnosis and treatment of DO. Meanwhile, anatomy morphology had also been intensively investigated within the development of surgery. In the field of pediatrics, the emphasis was placed on DO bone reconstruction surgery for the treatment of skeletal dysplasia in children. DO can correct skeletal deformities such as limb and head deformities in children, so as to promote the recovery of skeletal function and morphology in patients. Vogt et al. (2011) studied the use of DO technique to correct forearm deformities caused by multiple chondroectogenesis in children. With the progress of research experimental medicine, more and more studies have applied animal experiments or clinical trials to the pathology and physiology of DO. In addition, researchers combine physiology, pathology, and experimental medical research to explore the biological mechanism of DO. For example, Hamushan et al. (2021) demonstrated that high purity magnesium needles can promote bone growth by mechanical test, radiology and histological analysis of rat femur, which may be related to the regulation of Ptch protein activating Hedgehog pathway instead of Wnt signal transduction. Meanwhile, some studies have also shown that the growth hormone can promote callus regeneration in patients with X-linked hypophosphatemic rickets during DO (Canete et al., 2014). In the field of rehabilitation, it mainly focuses on the role of physical therapy (such as low-intensity pulse ultrasound, exercise therapy) in promoting callus growth and limb function recovery in the process of DO. El-Mowafi and Mohsen (2005) found that low-intensity pulsed ultrasound stimulation was effective in promoting tibia maturation and shortening bone formation time.

### 3.5 Bibliographic coupling analysis

#### 3.5.1 Country

**FIGURE 6:**
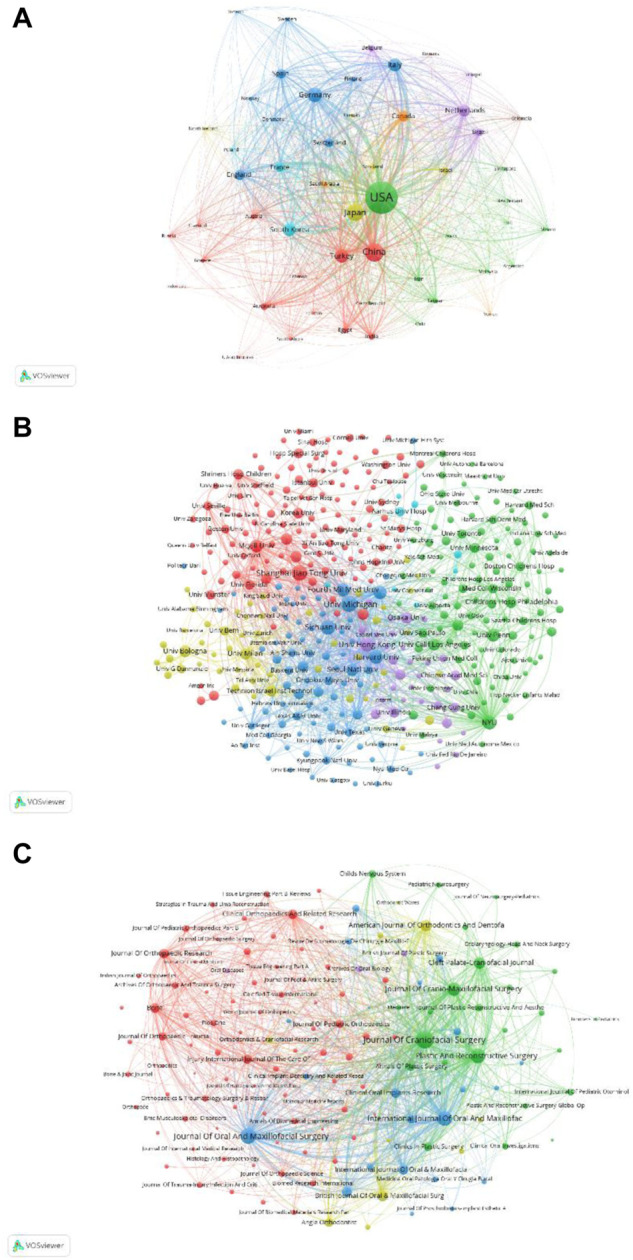
Bibliographic coupling analysis of global publications in DO in the last 42 years. **(A)** Network visualization of the 54 identified countries in DO. **(B)** Network visualization of the 399 identified institutions in DO. **(C)** Network visualization of the 141 identified journals in DO. In the visualized network, items are represented by circles. The larger the circle, the more the number of publications; the thickness of line represents the correlation strength; and the circle color represents different cluster.

Figure 6A presents the relevance of 54 identified countries (the minimum number of documents of a country more than 5). The top five countries with TLS are as follows: the United States (TLS = 1142009), China (TLS = 461186), Japan (TLS = 405909), Germany (TLS = 309495) and Turkey (TLS = 276394). Therefore, according to bibliographic coupling analysis, the United States is the leading country in DO around the world.

#### 3.5.2 Institution

Figure 6B shows the relevance of 399 identified institutions (the minimum number of documents of an institution is more than 5). The top five institutions with TLS are New York University (TLS = 103555), Harvard University (TLS = 99245), University of Michigan (TLS = 96046), University of Hong Kong (TLS = 85036) and McGill University of Canada (TLS = 84412). Therefore, through the above analysis, New York University is the leading institution of DO in the world.

#### 3.5.3 Journal

Figure 6C shows the relevance of 141 identified journals (the minimum number of documents of a journal is more than 5). The top five journals with TLS are as follows: Journal of Craniofacial Surgery (TLS = 406287), Journal of Oral And Maxillofacial Surgery (TLS = 268937), Plastic and Reconstructive Surgery (TLS = 231890), International Journal of Oral and Maxillofacial Surgery (TLS = 209614) and Journal of Cranio-Maxillofacial Surgery (TLS = 157150). Therefore, according to bibliographic coupling analysis, Journal of Craniofacial Surgery is the leading global journal in DO.

### 3.6 Co-authorship analysis

#### 3.6.1 Country

**FIGURE 7:**
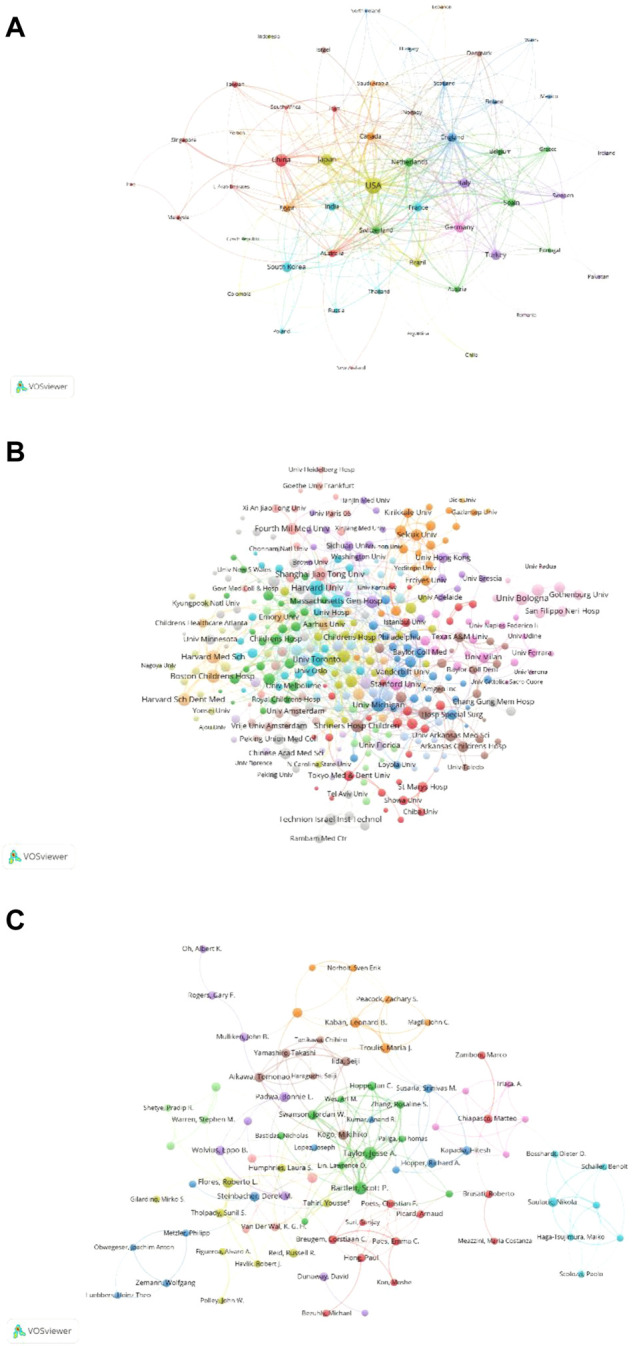
Co-authorship analysis of global publications in DO in the last 42 years. **(A)** Network visualization of the 53 identified countries in DO. **(B)** Network visualization of the 371 identified institutions in DO. **(C)** Network visualization of the 86 identified authors in DO.

Figure 7A presents the relevance of 53 identified countries (the minimum number of documents of a country is more than 5) in TLS. The top five countries with TLS are the United States (TLS = 399), the England (TLS = 163), Germany (TLS = 148), and Italy (TLS = 125), the Switzerland (TLS = 115). Therefore, according to the co-authorship analysis, American authors are more cooperative than their authors in other countries.

#### 3.6.2 Institution

Figure 7B shows the relevance of 371 identified institutions (the minimum number of documents of an institution is more than 5) in TLS. The top five institutions with TLS are Harvard University (TLS = 68), University of Bologna (TLS = 55), University of Michigan (TLS = 51), Boston Children’s Hospital (TLS = 50), Shanghai Jiaotong University (TLS = 48). Therefore, according to the co-authorship analysis, Harvard University is more cooperative than other universities.

#### 3.6.3 Author

Figure 7C shows the relevance of 86 identified author (the minimum number of documents of an author is more than 5) in TLS. The top five authors are as follows: Bartlett, Scott P. (TLS = 92), Taylor, Jesse A. (TLS = 88), Swanson, Jordan W. (TLS = 53), Hoppe, Ian C. (TLS = 41), and Zhang, Rosaline S. (TLS = 39). Therefore, according to the co-authorship analysis, Bartlett, Scott P. is the most cooperative author.

### 3.7 Co-citation analysis

#### 3.7.1 Journal

**FIGURE 8:**
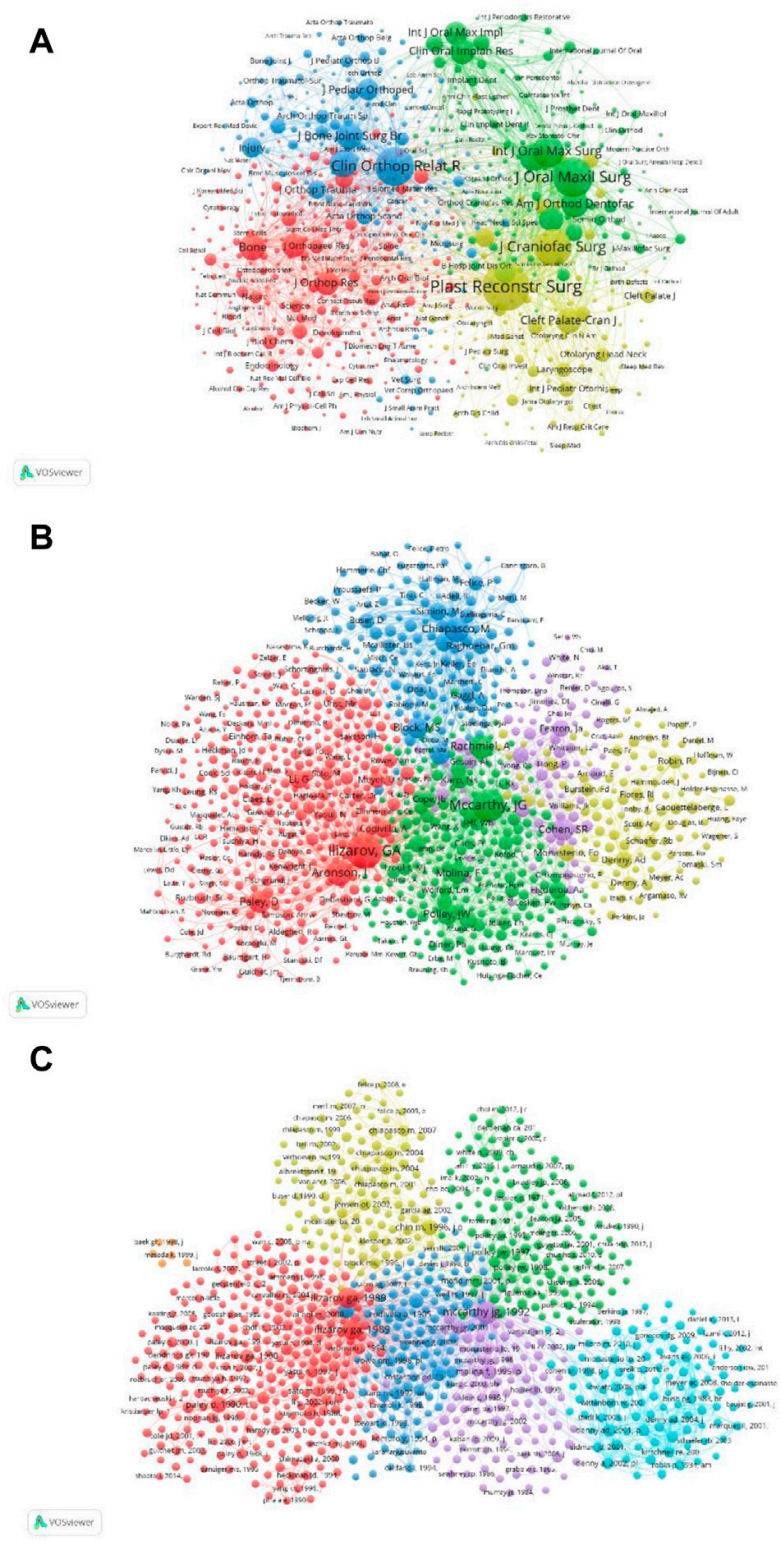
Co-citation analysis of global publications in DO in the last 42 years. **(A)** Network visualization of the 712 identified journals in DO. **(B)** Network visualization of the 1257 identified authors in DO. **(C)** Network visualization of the 906 identified references in DO.

Figure 8A shows the relevance between the TLS of 712 identified journals (the minimum number of citations of a journal is more than 20). The top five journals with TLS are as follows: Plast Reconstr Surg (TLS = 427555), J Oral Maxil Surg (TLS = 355213), J Craniofac Surg (TLS = 273571), Clin Orthop Relat R (TLS = 269309) and Int J Oral Max Surg (TLS = 201085). Therefore, according to the co-citation analysis, Plast Reconstr Surg is the influential journal of DO in the world.

#### 3.7.2 Authors

Figure 8B reveals the relevance between the TLS of the 1257 identified authors (the minimum number of references to a publication is more than 20). The top five publications with TLS are Ilizarov GA (TLS = 42691), Mccarthy JG (TLS = 34527), Aronson J (TLS = 18810), Chiapasco M (TLS = 17265), and Rachmiel A (TLS = 15678). Therefore, according to the co-citation analysis, Ilizarov GA are the most influential authors of DO in the world.

#### 3.7.3 References

Figure 8C reveals the relevance between the TLS of the 906 identified publications (the minimum number of references a publication is more than 20). The top five publications with TLS are as follows: McCarthy et al. (1992b) (TLS = 11983), Ilizarov (1989b), Ilizarov (1989c) (TLS = 9124 and 8692), Molina and Ortiz Monasterio (1995) (TLS = 3959) and Chin and Toth (1996) (TLS = 3875). Therefore, according to the co-citation analysis, The paper by McCarthy et al. (1992b) is the most influential publications of DO in the world.

### 3.8 Co-occurrence analysis

**FIGURE 9:**
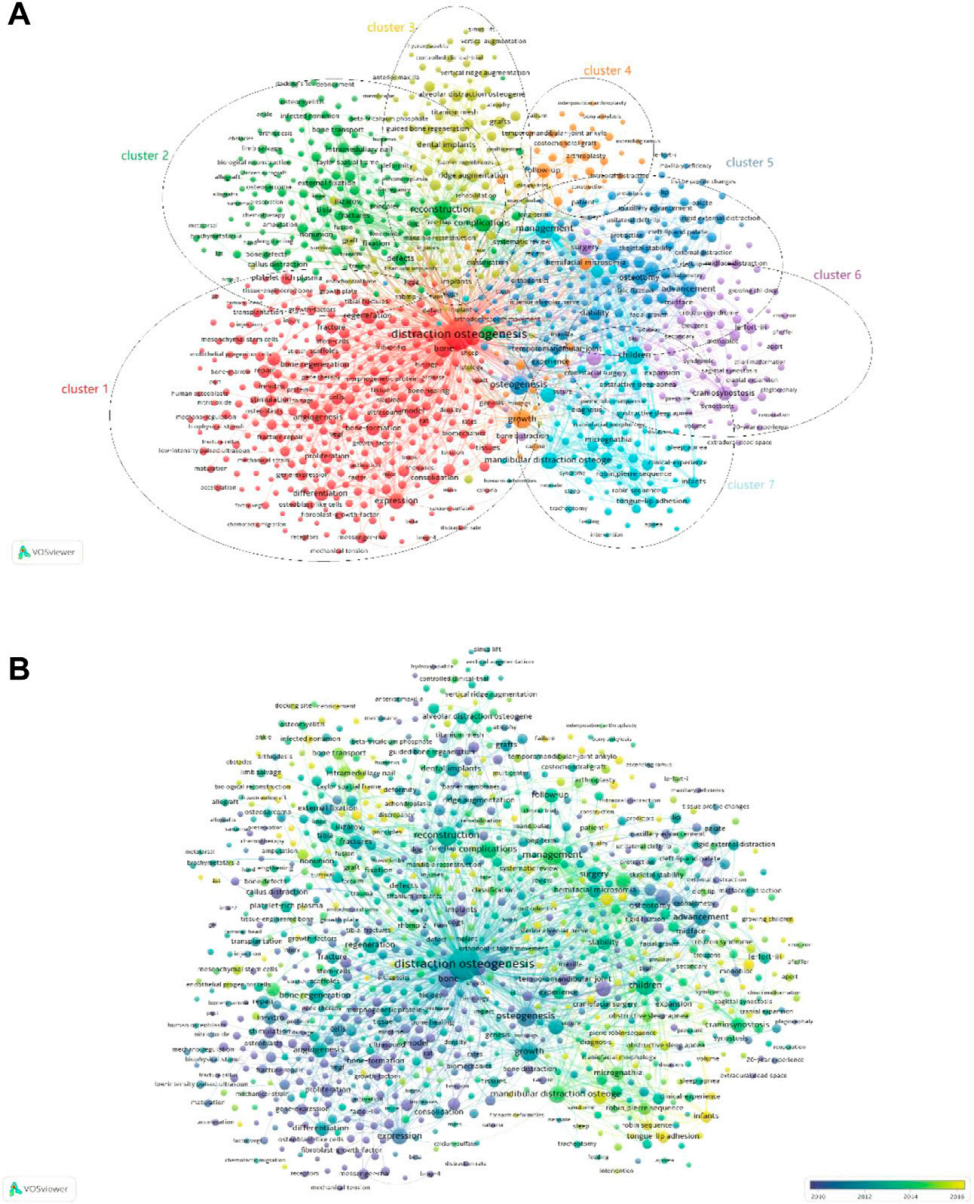
Co-occurrence analysis of global publications in DO in the last 42 years. **(A)** Network visualization of 1000 identified keywords in DO. All the keywords are divided into 7 clusters: 1) “mechanism study”, 2) “limb bone distraction study”, 3) “alveolar bone distraction study”, 4) “temporomandibular joint ankylosis study”, 5) “maxillofacial surgery study”, 6) “skull distraction study” and 7) “mandible distraction study”. **(B)** Overlay visualization of the identified 662 keywords in DO based on the average time they appeared in the publications. The blue keyword appeared earlier, while the yellow keyword appeared later.

As shown in Figure 9A, 1,000 identified keywords (the minimum number of occurrences of a keyword in titles and abstracts is more than 10) are divided into 7 clusters: “mechanism study”, “limb bone distraction study”, “alveolar bone distraction study”, “temporomandibular joint ankylosis study”, “maxillofacial surgery study”, “skull distraction study” and “mandible distraction study”. The top 10 keywords in DO are “distraction osteogenesis”, “management”, “osteogenesis”, “reconstruction”, “growth”, “children”, “complications”, “bone”, “mandibular distraction osteogenesis,” and “expression”. These results can provide new insights into the hot research directions and topics of DO, which indicates that further attention should be paid to these promising areas in the future.

The overlay visualization can give items different colors depending on the average time of keyword appearance. The bluer the color, the earlier the keyword appears, the more yellow the color, the later the keyword appears (Figure 9B). By comparing Figure 9A with Figure 9B, we can find that the cluster 7 region is more yellow in Figure 9B, which means that the keywords related to mandibular distraction appeared later. Therefore, “mandible distraction study” is a hot topic of DO-related research in recent years and may become the focus of future research. While before 2012, most studies focus on “mechanism study.”

## 4 Strengths and limitations

Our study employs bibliometric research methods to provide a comprehensive and objective picture of global trends and the current state of DO-related publications from 1980 to 2021. However, the study also has some limitations. First, the study measures the quality of publications primarily by comparing the number of citations and the average times of citations for per item. However, highly cited publications do not equate to high scientific quality. The number of citations can be influenced by some factors, such as the hot issue of research topic, fame degree of researcher, or even over-cited artificially. Secondly, there are differences in publications from different databases, and we used only the WOS core collection database and Medline for literature data search, which may have an impact on the accuracy of the study results. Finally, the influence of publication time on the total number of citations was not considered. The fact that the most recent high-quality articles are rarely cited may be overlooked. Therefore, it is also important to pay attention to the most recent publications.

## 5 Conclusion

Our study identifies DO-related publications from 1980 to 2021 and introduces their global trends and status. In the past 42 years, the number of total publications of DO research has maintained a steady trend after rapid growth. The United States ranks first in terms of total publications, sum of times cited, the H-Index and self-citation times. Harvard University, University of California and Egyptian Knowledge Bank are the top 3 contributing institutions to DO. Journal of Craniofacial Surgery, Journal of Oral and Maxillofacial Surgery and Plastic and Reconstructive Surgery are the top 3 contributing journals to DO. Buchman SR, Mccarthy JG and Cheung LK are the top 3 contributing authors to DO. Surgery, orthopaedics and anatomy morphology are the top 3 fields of DO research. In addition, the DO research on the mandible will become the focus of scholars in the future.

## Data Availability

The original contributions presented in the study are included in the article/supplementary material, further inquiries can be directed to the corresponding authors.
